# Analgesic efficacy of ultrasound-guided bilateral transversus abdominis plane block in children: retrospective analysis of 97 cases

**DOI:** 10.55730/1300-0144.5594

**Published:** 2023-01-04

**Authors:** H. Kutluk PAMPAL, Selin EREL, Semin TURHAN, Asiye UĞRAŞ DİKMEN, Berrin IŞIK

**Affiliations:** 1 Department of Anesthesiology and Reanimation, Gazi University, Ankara, Turkey; 2 Department of Anesthesiology and Reanimation, Hitit, University, Çorum, Turkey; 3 Department of Public Health, Gazi University, Ankara, Turkey

**Keywords:** Anesthesia, general anesthesia, nerve block, postoperative pain, pediatrics

## Abstract

**Background/aim:**

Transversus abdominis plane (TAP) block is a method for postoperative pain management. Studies on children are gradually increasing. The aim of this retrospective study was to evaluate effectiveness of TAP block on pain control, its side effects, and parental satisfaction levels in children.

**Material and methods:**

Study included patients operated between January 2019 and December 2020 in Gazi University Faculty of Medicine. Total of 97 patients (35 girls, 62 boys) between 5 and 18 years who had an ultrasound guided TAP block for lower abdominal or inguinal surgery were examined retrospectively. TAP block application time, hemodynamic variables, postoperative pain scores, postoperative analgesic requirement, sex, surgical history and satisfaction levels were evaluated.

**Results:**

The average application time of TAP block was 9.48 ± 3.4 and the time between TAP block and surgical incision was 12.06 ± 6.1 min. Pain scores in postanesthesia care unit (PACU) and at the postoperative first hour decreased as the time between TAP block and surgical incision increased (p < 0.05). Girls have higher pain scores at PACU than boys (p < 0.05). Previous surgical history increased postoperative 1st hour pain scores (OR: 13.8; 95% CI 1.7–113.3; p = 0.01). There was a significant negative correlation between pain scores at PACU, postoperative 1st, 2nd, 4th, 6th, 12th and satisfaction levels (r = −0.45, r = −0.56, r = −0.60, r = −0.54, r = −0.52, r = −0,43, respectively, p < 0.05).

**Conclusion:**

Ultrasound-guided TAP blocks can be performed safely in children in lower abdominal surgeries. However, the efficacy of TAP block on late term postoperative pain scores is limited. Time interval between the TAP block and the incision, sex, and pain memory, as well as other factors that may improve the quality of TAP block should be considered.

## 1. Introduction

In pediatric age groups, the treatment of postoperative pain has more concerns than in adults. Opioids are used for postoperative analgesia. However, side effects hinder their utility in children [1]. After surgery, a wide proportion of children receive insufficient analgesic [2]. Due to its efficient pain management and the enhanced safety profile of the local anesthetic agent with the guidance of ultrasound, the use of pediatric regional anesthesia has increased [1]. Transversus abdominis plane (TAP) block and its modifications such as ultrasound guided TAP block are one of the regional techniques that is getting more applied in children [3, 4].

The TAP block provides a local anesthetic drug diffusion between transversus abdominis and obliquus internus abdominis muscles. The block reaches the anterior rami of spinal nerves from T7 to L1 involved within the innervation of the abdominal wall [5]. It provides cutaneous sensory block predominantly lateral to vertical line through the anterior superior iliac spine, and its distribution is nondermatomal and does not cross the midline [6]. Although the efficiency of it for perioperative pain control has been demonstrated, contradictory results are still being reported. Studies comparing TAP block to other regional blocks, such as caudal epidural, ilioinguinal, or quadratus lumborum reveal increased postoperative analgesic consumption in TAP block groups [7, 8].

In this retrospective study, we aimed to assess the impact of TAP block in terms of postoperative analgesia, side effects, and patient’s or parent’s satisfaction in 5 to 18 years old children who underwent lower abdominal or inguinal surgery.

## 2. Material and methods

After obtaining ethical approval from Gazi University Clinical Research Ethics Committee (no: 281, date: 27/04/2020), retrospective evaluation of the anesthesia records of patients <18 years old underwent lower abdominal or inguinal surgery, with an ultrasound-guided TAP block performed under general anesthesia within 2019 to 2020 were conducted. The data retained by the Departments of Pediatric Anesthesiology, and the Department of Pediatric Surgery medical charts of the patients were reviewed.

A total of 141 patient files were accessed. Forty-four patients were not included in the study due to missing data or if they had received TAP block at the end of the surgery. This retrospective study involved 97 children of both sexes, aged 5–18 years, weighing 16–100 kg, who received general anesthesia for lower abdominal or inguinal surgery. All children were anesthetized using the standard protocol (general anesthesia induction consisted of propofol 2–3 mg/kg, remifentanil infusion 0.05 to 0.2 μg/kg/min and/or rocuronium 0.6 mg/kg followed by tracheal intubation or laryngeal mask airway insertion and mechanical ventilation). 0.8–1.2 minimum alveolar concentration of sevoflurane in oxygen/air mixture was administered for the maintenance of anesthesia. After that, TAP block was performed under general anesthesia. The ultrasound (Sonosite Edge II, Bothell, WA) guided technique was used to identify fascia between the transversus abdominis and internal oblique muscles and assess the spreading of local anesthetic solution. Under aseptic technique and with the patient in the supine position, probe was placed on the abdominal wall at the level of T10 dermatome. Rectus abdominis muscle was visualized then probe was moved to iliac crest and costal margin distance. Musculofacial layers of the abdominal wall were visualized, and local anesthetic was injected to TAP after negative aspiration to avoid intravascular injection. TAP block was performed bilaterally with 0.25% concentration bupivacaine (Bustesin 0.5% 20 mL Vem Pharmaceuticals, İstanbul/Turkey) by the same experienced pediatric anesthetist (B.I.). Total local anesthetic amount was limited to maximum of 0.2 mL/kg according to our hospital’s pediatric anesthesia unit postoperative pain treatment protocols. Besides all patients received intravenous acetaminophen (Parol 10 mg/mL Atabay, İstanbul/Turkey) 15 mg/kg at the end of the surgery. Patients were admitted to postoperative care unit (PACU) after surgery. Pain assessment was done using numeric rating scale (NRS) (0 to 10) if the child was older than 8 years old or cooperative [9]. If child was younger than 8 years old or noncooperative Faces Legs Activity Cry Consolability Revised Scale (FLACC-R) was used [9]. Rescue analgesics (i.v. morphine 0.05 mg/kg) were added in case of pain score >4 at PACU or ward. Patients were discharged to the ward when Aldrete score ≥ 9 [10].

Demographic properties, history of previous surgery, type, and length of surgery, anesthetic agents, and doses, local anesthetic concentration, and doses for TAP block, the processing time of TAP block, time from TAP block to surgical incision were evaluated.

Postoperative rescue analgesic need for analgesia and its time were assessed from medical records of patients. Based on these records mean arterial pressure (MAP) and heart rate (HR) were recorded in the following time points: preoperative, intraoperative 5th, 10th, 30th, 60th minutes, postoperative at PACU, 10th, 20th minutes, 1st, 2nd, 4th, 6th, 12th hours.

Pain scales were assessed immediately after admission to PACU and then at 1st, 2nd, 4th, 6th, and 12th hours after surgery. The level of satisfaction of the patient or their parents was assessed with a 5-point Likert Scale at the 12th hour. General anesthesia or TAP block related complications were assessed.

### 2.1. Statistical analysis

Statistical analysis was performed using the SPSS version 23 (Statistical Package for Social Sciences, IBM Corporation, Armonk, NY, USA) software. Categorical variables are presented as numbers and percentages, while continuous variables are presented as mean ± standard deviation (SD) and median (minimum-maximum). The compliance of continuous variables to normal distribution was evaluated using visual (histogram and probability graphs) and analytical methods (Kolmogorov-Smirnov/Shapiro-Wilk tests). Mann-Whitney U test was used for data that did not comply with the normal distribution and independent samples t-test was used normally distributed data. The Pearson chi-square test was used to compare categorical variables between independent groups. Pearson correlation test was used for correlation analysis. Univariate logistic regression analysis was performed to identify the associations between the sex and past surgical history and postoperative pain scores. The receiver operating characteristic (ROC) analysis was used to determine a cut-off value for deciding a sufficient time interval between the TAP block and surgical incision to prevent pain scores of ≥4. Significance level was set at p < 0.05.

## 3. Results

The study includes 97 patients’ data with ASA I-II, 35 girls, and 62 boys, 5 to 18 (mean 12.87 ± 3.61) years old. Demographic and TAP block-related variables were shown in Table 1. The mean application time for the TAP block was 9.48 ± 3.47 min. For the TAP block, an average of 0.6 mL/kg bupivacaine was used. Perioperative MAP and HR values were presented in Table 2.

Assessment of the relationship between hemodynamic parameters and pain revealed a correlation between pain scores and MAP but not HR. First hour pain score and intraoperative 5th, 10th and postoperative 10th minutes MAP values were positively correlated (r = 0.27, p = 0.006; r = 0.20, p = 0.004; r = 0,29, p= 0.006). Also, a positive correlation was observed between 4th hour pain score and postoperative 10th minute MAP (r = 0.22, p = 0.032).

Rescue analgesia requirement, satisfaction levels, and postoperative pain scores are shown in Table 3. There was a positive correlation between pain scores in the PACU and the pain scores at 1st, 2nd, 4th, and 6th hours (r = 0.55, p < 0.001; r = 0.37, p < 0.001; r = 0.29, p < 0.003; r = 0.22, p =0.028). This association was not detected at the 12th hour postoperatively.

As the time between the TAP block application and the beginning of the surgery increased, the recovery and first hour pain scores decreased (p < 0.001, p = 0.01, respectively). A Pearson’s r data analysis revealed a moderate negative correlation, r = −0.62 and −0.26, respectively. Time interval between the TAP block and surgical incision did not affect pain scores at the 2nd, 4th, 6th, and 12th hours.

ROC analysis revealed a cut-off value of 12.5 min for the duration of the interval between TAP block and surgical incision can predict pain scores higher than 4 with a sensitivity of 0.90 and a specificity of 0.73 for pain score at PACU and with a sensitivity of 0.62 and a specificity of 0.65 for 1st hour pain score. The ROC analysis for time interval between TAP block and surgical incision was presented in Table 4.

In terms of postoperative pain scores at PACU and 1st hour, the pain scores were higher in the girls than the boys (p = 0.001 and p = 0.031, respectively) (Figure). The mean pain score in PACU was 6.20 ± 2.34 for girls while it was 4.21 ±2.58 for boys.

Univariate logistic regression analysis revealed that previous surgical history increased postoperative 1st hour pain scores (OR: 13.8; 95% CI 1.7–113.3; p = 0.01).

There was no difference in terms of postoperative satisfaction levels between patients who have previously undergone surgery and those who were operated for the first time. An inverse correlation between pain scores at all time intervals ​​and satisfaction levels was detected. Satisfaction increased as the pain scores decreased (p < 0.001). A negative correlation between pain scores at PACU, postoperative 1st, 2nd, 4th, 6th, 12th and satisfaction levels were observed (r = −0.45, r = −0.56, r = −0.60, r = −0.54, r = −0.52, r = −0,43, respectively, p < 0.05).

The reviewed anesthetic, surgical and follow-up forms at the ward revealed no TAP block and general anesthesia related complications.

## 4. Discussion

The most notable findings of this retrospective study are; (i)TAP block is more effective in boys than in girls, (ii) past surgical experience in our patient population had a negative impact on the analgesic effect of TAP block, (iii) the quality of TAP block weakens, and pain scores increase when the time interval between TAP block and incision is insufficient.

Sex variations in perception and evaluation of pain have been widely studied. The studies do not fully cover pediatric population and the results are still conflicting [11]. The pain scores may differ based on the patients’ race and sex. Studies do not indicate a single cause for the disparity in the sex-based measurement of pain. Many factors may cause differences, such as biological physiology and differences in socialization. Girls are more distressed than boys and have higher postoperative pain scores [12, 13]. Parallel to the studies, significant differences in pain ratings between sexes were noted in our study. The mean pain scores of the female sex were higher than males.

Pain memory is one of the key reasons why children who had undergone past surgery have higher pain scores in the course of the repetitive procedures. Hyperalgesia due to spinal and supraspinal changes caused by tissue damage in early life may lead to aforementioned phenomena [14, 15]. Prolonged pain hypersensitivity has also been considered as another possible mechanism [15]. Higher pain scores of the children who had previous surgical intervention in the current study also supports the results of the previous studies mentioned. However, further experimental, and clinical studies should be performed in order to fully understand the exact mechanism.

Preemptive analgesia is the administration of an analgesic prior to noxious surgical stimulation. Metaanalyses have revealed different outcomes regarding the efficacy of preemptive analgesia on postoperative pain management [16, 17]. We observed that TAP block’s ability to lessen postoperative pain, improved with the increased time interval between surgical incision and the block. When choosing TAP block for preemptive analgesia, it is better to ensure enough time between the onset of the block and the painful stimulus. Effective analgesia cannot be provided when the time between the block and the incision is insufficient. In the early postoperative period, patients with NRS < 4 have still had low pain scores in the following hours.

Transversus abdominis plane block has been an accepted part of multimodal analgesia and has been used in a wide variety of procedures, in the pediatric population for lower abdominal surgeries such as herniotomy, laparoscopy, appendectomy, laparotomy, and colostomy [18–20]. With limited complications in the pediatric age group, ultrasound guided TAP blocks have promising effects and suggested as feasible alternative to neuraxial blocks in case when there are contraindications such as coagulopathy, spinal deformity, etc. [19, 21].

Transversus abdominis plane block appears to have a valuable role in patients with comorbidities and give rise to hope, especially when central neuraxial blocks are contraindicated. Applying the TAP block alone in awake adult surgical patients offers a chance to detect complications at an early stage. However, in smaller children, TAP block is performed under general anesthesia. Unnoticing complications arising from the procedure or local anesthetics are the main risk. As local anesthetic pharmacokinetics differ in children compared to adults, this factor makes it difficult to determine nontoxic dose ranges [22]. Based on the literature, the TAP block appears to be safe, with minor complications (up to 0.3%) requiring no intervention [23]. But rarely intravascular injection, local anesthetic systemic toxicity, liver or spleen laceration have been reported [24–27]. In the study, none of the 97 patients had any major or minor complications.

Indications of TAP block and patient population in this study were similar to the literature [18, 19]. We performed TAP block for patients undergoing appendectomy, inguinal hernia, varicocele, ovarian cyst, undescended testis, hypospadias, circumcision, hydrocele, mesenteric cyst and orchiectomy, surgeries. Similar to the studies that have negative results for TAP block, our study also did not show much benefit on postoperative pain scores. Pain scores at PACU, 1st and 2nd postoperative hours were shown to be high. Despite the block, 53% of the patients in the PACU, 40% at the 1st hour, and 33% at the 2nd hour were found to have pain scores > 4. It indicates that effective pain control could not be achieved, and rescue analgesics were required.

The entry points of the T6-L1 nerves are highly variable; therefore, some techniques cannot adequately block the related nerves [28]. Subcostal and lateral TAP injections do not often cover the lateral cutaneous branches of the segmental nerves, the posterior injection can provide improved analgesia to the lateral abdominal wall [29]. Since lateral approach is the choice of technique in our clinic, the high pain scores in children underwent abdominal surgery under general anesthesia in our study can be attributed to the technique we used.

In our research, we applied bilateral TAP block under ultrasound with a lateral approach. The block was performed with 0.25% concentration bupivacaine with a limit dose of 0.2 mL/kg. The mean dose of bupivacaine applied per kg was found to be 0.6 mg. Optimal dosage, volume, and kind of local anesthetic for TAP blocks are still undetermined according to the latest research, yet total dose of bupivacaine is also limited; to 2 mg/kg in neonates, 3 mg/kg in children, and 4 mg/kg in adolescents to avoid local anesthetic toxicity [21]. In this context, the dose we used in the TAP block may have been insufficient. With higher volumes and concentrations, more efficient analgesia can be achieved. Our concern about reaching toxic dose with local anesthetics in pediatric age group may have caused insufficient administration of the drug [22]. In addition to the previous possible mechanisms, the analgesic property of TAP block as it only prevents somatic pain but not visceral, may contribute to the failure of TAP block in our study population [21].

Acute pain generates an increasing sympathetic nerve activity. The initial response to nociceptive stimuli is the activation of some components of the sympathetic nervous system. Substances such as adrenocorticotrophic hormone, glucocorticoids, epinephrine and norepinephrine, etc., are released. Reaction to these mediators involves changes in cardiovascular, endocrine, somatosensory systems which maintains physiological functions against the imbalance due to surgical stress [30, 31]. According to our study, patients with higher postoperative pain scores also had high perioperative MAP values. This suggests that a strong link may exist between early sympathetic response and the ineffectiveness of the TAP block applied to prevent pain.

### 4.1. Limitations of study

The analysis of this data has number of limitations. The retrospective design restricts the study’s ability to test for confounding intraoperative factors. Since we administered morphine as a rescue analgesic when the pain scores was ^3^4 for ethical reasons, it was not possible to evaluate the late effects of solely TAP block in these cases.

## 5. Conclusion

In accordance with preemptive analgesia principles, the surgical team involved in the treatment of these patients must treat cautiously in terms of adequate timing between the TAP block and surgical incision. Sex differences and pain memory, in addition to other factors that may improve the quality of TAP block, should be considered during postoperative pain management with TAP block. Also, it is critical to use the highest amount of local anesthetic drug possible when performing TAP block.

## Figures and Tables

**Figure:**
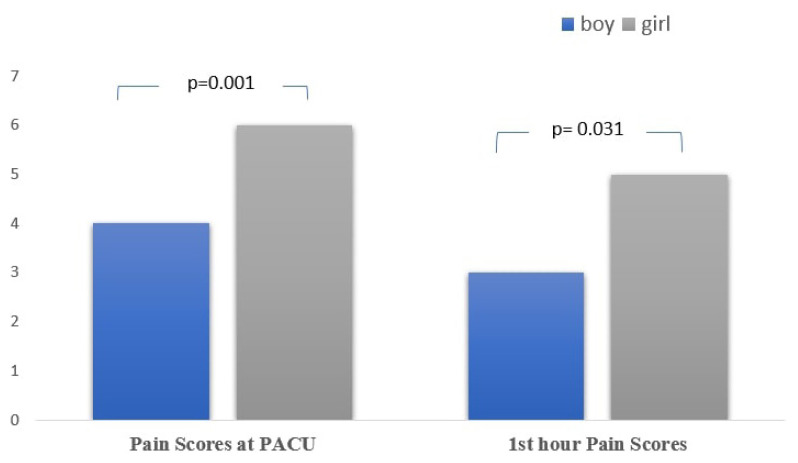
Sex and pain scores at PACU and postoperative 1st hour pain score.

**Table 1:** Demographic variables, history of surgery, induction of anesthesia, TAP block, and surgery data (n, %, min-max, mean ± SD).

Sex	
*Male/Female (n, %)*	62 (63.9)/35 (36.1)
**Age (year)** mean ± SD	12.87 ± 3.6
**Body weight (kg)** mean ± SD	50.92 ± 17.3
**Type of surgery** (n, %)	
*Appendectomy*	50 (51.5)
*Inguinal hernia*	15 (15.5)
*Varicocele*	12 (12.4)
*Ovarian cyst*	8 (8.2)
*Undescended testis*	3 (3.1)
*Hypospadias*	3 (3.1)
*Circumcision*	2 (2.1)
*Hydrocele*	2 (2.1)
*Mesenteric cyst*	1 (1.0)
*Orchiectomy*	1 (1.0)
*Appendectomy*	50 (51.5)
**History of previous surgery**	
*Absent (n, %)*	86 (88.7)
*Present (n, %)*	11 (11.3)
**Type of previous surgery**	
*Ear nose and throat surgery*	4 (4.1)
*Lower abdominal surgery*	4 (4.1)
*Cranial surgery*	1 (1.0)
*Cardiac surgery*	1 (1.0)
*Orthopedic surgery*	1 (1.0)
**Time of surgery (min)** mean ± SD	61.56 ± 20.8
**TAP block processing time (min)** mean ± SD	9.48 ± 3.4
**Time between TAP block and surgical incision (min)** mean ± SD	12.06 ± 6.1

**TAP:** transversus abdominis plane; **min**: minute.

**Table 2:** Preoperative MAP and HR values of the patients.

	MAP	HR
Preoperative	77.9 ± 13.3	93.3 ± 17.4
Intraoperative 5th min	70.0 ± 13.1	84.9 ±16.0
Intraoperative 10th min	70.5 ± 12.6	82.0 ± 17.2
Intraoperative 30th min	70.9 ± 11.0	79.6 ± 16.3
Intraoperative 60th min	71.2 ± 11.2	78.0 ± 13.4
PACU	87.3 ± 12.5	94.7 ± 19.9
Postoperative 10th min	85.5 ± 10.1	86.4 ± 13.7
Postoperative 20th min	82.5 ±10.9	83.3 ± 12.7
Postoperative 1st h	83.1 ± 9.1	90.9 ± 19.3
Postoperative 2st h	78.8 ± 9.1	85.9 ± 13.9
Postoperative 4st h	77.8 ± 9.7	85.3 ± 14.9
Postoperative 6st h	79.2 ± 9.2	81.1 ± 12.8
Postoperative 12st^t^ h	78.3 ± 8.8	76.0 ± 11.5

**MAP**: mean arterial pressure; **HR**: heart rate; **min**: minute; **PACU**: postanesthesia care unit; **h**: hour.

**Table 3:** Rescue analgesic need (n/%), satisfaction levels (n/%) and postoperative NRS variables (min-max).

Rescue analgesic	(n, %)
*Need*	60 (61.9)
*No need*	37 (38.1)
**5-point Likert satisfaction scale**	**(n, %)**
*Very satisfied*	19 (19.6)
*Satisfied*	35 (36.1)
*Neutral*	27 (27.8)
*Dissatisfied*	11 (11.3)
*Very dissatisfied*	5 (5.2)
**Postoperative NRS values**	**Mean ± SD**
*PACU*	4.93 ± 2.6
*1st hour*	4.18 ± 2.4
*2nd hour*	3.41 ± 2.4
*4th hour*	2.86 ± 2.4
*6th hour*	1.92 ± 2.0
*12th hour*	0.32 ± 1.7

**PACU**: postanesthesia care unit, **NRS**: numeric rating scale.

**Table 4:** Area under the curve, sensitivity and specificity by the cut-off point for time interval between TAP block and surgical incision.

Pain assessment points	AUC (95 %CI)	Cut-off	p	Sensitivity	Specificity
PACU	0.82(0.74–0.91)	12.5 min	**<0.001**	0.90	0.73
1st h	0.65 (0.53–0.76)	12.5 min	**0.01**	0.62	0.65
2nd h	0.56 (0.44–0.68)	N/A	0.31	N/A	N/A
4th h	0.53 (0.40–0.65)	N/A	0.62	N/A	N/A
6th h	0.68 (0.55–0.81)	N/A	0.05	N/A	N/A
12th h	0.71 (0.57–0.86)	N/A	0.05	N/A	N/A

**TAP:** transversus abdominis plane; **AUC**: area under the curve; **PACU**: postanesthesia care unit; **h**: hour; **min**: minute.
